# Dietary pattern and hepatic lipid metabolism^☆^

**DOI:** 10.1016/j.livres.2023.11.006

**Published:** 2023-11-29

**Authors:** Peng Zou, Lin Wang

**Affiliations:** Department of Hepatobiliary Surgery, Xi-Jing Hospital, The Fourth Military Medical University, Xi’an, Shaanxi, China

**Keywords:** Non-alcoholic fatty liver disease (NAFLD), Hepatic lipid metabolism, Dietary pattern, Mediterranean diet, Ketogenic diet

## Abstract

The liver is the leading site for lipid metabolism, involving not only fatty acid beta-oxidation but also *de novo* synthesis of endogenous triglycerides and ketogenesis. The liver maintains systemic lipid homeostasis by regulating lipid synthesis, catabolism, and transportation. Dysregulation of hepatic lipid metabolism precipitates disorders, such as non-alcoholic fatty liver disease (NAFLD), affecting the whole body. Thus, comprehending and studying hepatic lipid metabolism is crucial for preventing and treating metabolic liver diseases. Traditionally, researchers have investigated the impact of a single nutrient on hepatic lipid metabolism. However, real-life dietary patterns encompass diverse nutrients rather than single components. In recent years, there have been increased studies and notable progress regarding the effects of distinct dietary patterns on hepatic lipid metabolism. This review summarizes the influence of diverse dietary patterns on hepatic lipid metabolism, elucidating underlying molecular mechanisms and appraising the therapeutic potential of dietary patterns in managing hepatic steatosis.

## 1 Introduction

The liver is an organ where various chemical reactions occur and plays a significant role in maintaining lipid metabolism. These reactions include glycogenesis, glycogenolysis, gluconeogenesis, lipid synthesis, and fatty acid oxidation. Hepatic lipid metabolism comprises cholesterol metabolism, fatty acid oxidation, lipoprotein metabolism, and triglyceride (TG) metabolism. The imbalance of this metabolism can lead to health problems such as non-alcoholic fatty liver disease (NAFLD) and obesity.^1^ NAFLD has become the most prevalent chronic liver disease in Europe and the United States, with its prevalence gradually increasing globally.^2^ NAFLD is not a single disease but a spectrum of diseases that ranges from steatosis in the liver to non-alcoholic steatohepatitis (NASH) with varying degrees of fibrosis and cirrhosis.^3^ NASH refers to hepatic steatosis with lobular inflammation and hepatocyte injury after the progression of isolated steatosis, which is a major cause of cirrhosis and hepatocellular carcinoma.^4^^,^^5^

**Fig. 1:**
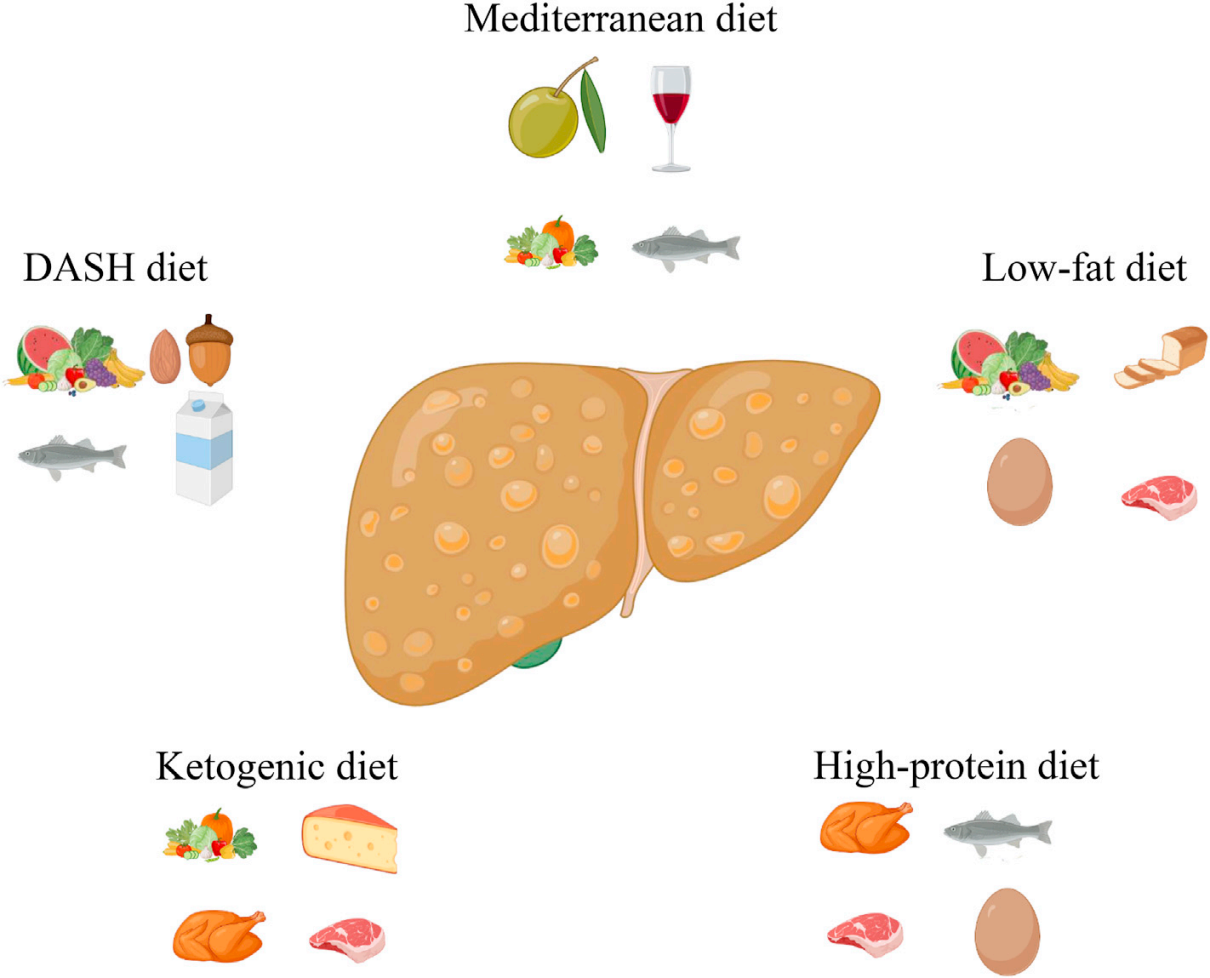
**Different dietary patterns**. This diagram depicts the foods that are representative of the five dietary patterns. This figure was created with BioRender.com. Abbreviation: DASH, Dietary Approaches to Stop Hypertension.

As there is currently no effective drug treatment for NAFLD, there is increasing interest in the therapeutic effect of lifestyle interventions on NAFLD, and dietary changes are an integral part of lifestyle interventions.^6^, ^7^, ^8^ Dietary changes involve not just adjusting individual nutrients but also altering the overall nutrient composition and proportion, which involves a modification of the dietary pattern. The Mediterranean diet is widely regarded as an ideal dietary pattern for people with NAFLD.^9^ However, due to variations in eating habits and food resources across different regions, promoting the Mediterranean diet universally is challenging, making scientists explore more reasonable dietary patterns for people worldwide. Therefore, this review will focus on the relationship between different dietary patterns (Fig. 1 and Table 1) and hepatic lipid metabolism, as well as the role of dietary patterns in the development of NAFLD.

**Table 1:** Dietary features and components of different dietary patterns.

Dietary patterns	Dietary features and components
Mediterranean diet	Extra virgin olive oil, fresh vegetables, fruit, unprocessed cereal, nuts, various legumes, intermediate intake of fish and other meat, red wine, and low consumption of eggs and sweets
DASH diet	Low sodium intake, low saturated fat, and high level of fiber and potassium: fruits, vegetables, cereal, and fish
Ketogenic diet	Low carbohydrates, low protein, and high fat: vegetables, chicken, meat, dairy products, and milk
Low-fat diet	Low fat and relatively high carbohydrates: fruit, egg, bread, white meat, seafood, fish, and red meat
High-protein diet	Proportion of protein, 25%–35%; carbohydrate, <45%

Abbreviation: DASH, Dietary Approaches to Stop Hypertension.

## 2 Mediterranean diet and hepatic lipid metabolism

The Mediterranean diet originated in countries along the Mediterranean coast, such as Greece, Italy, and Crete, and was first studied by Ancel Keys and Francisco Grande during the 1960s.^10^ In a 25-year study involving seven countries, this diet was associated with a reduced incidence of coronary heart disease; thus, it is gradually gaining attention.^11^ Although this dietary pattern varies in different countries, such as Italy, Albany, Spain, France, Lebanon, Morocco, Portugal, Syria, Tunisia, and Turkey, the main components of the Mediterranean diet remain consistent. They include cold pressed extra virgin olive oil as the dominant fat source, seasonal fresh vegetables, fruit, unprocessed cereal, nuts, various legumes, intermediate intakes of fish and other meat, dairy products, and red wine, and low consumption of eggs and sweets.^12^, ^13^, ^14^ Since then, several studies have provided evidence for the health benefits of the Mediterranean diet. Numerous studies have consistently demonstrated that the Mediterranean diet is associated with a significant reduction in overall mortality,^15^, ^16^, ^17^ a lower incidence of cardiovascular disease (CVD), improved CVD outcomes,^17^, ^18^, ^19^ a reduced risk of diabetes, enhanced efficacy in glycemic control for individuals with diabetes,^20^^,^^21^ a notable decrease in cancer incidence and mortality like breast cancer, colorectal cancer, liver cancer, head and neck cancer, and gastric cancer,^22^^,^^23^ as well as a lower risk of cognitive dysfunction, Parkinson’s disease, and Alzheimer’s disease.^15^^,^^24^^,^^25^

In addition to the above findings, a growing body of research has shown that the Mediterranean diet is associated with hepatic lipid metabolism. Montemayor *et al*.^26^ conducted a cohort study and found that the Mediterranean diet reduces hepatic fat content, liver stiffness, and TG levels and increases high-density lipoprotein (HDL) after 6 and 12 months of intervention. Moreover, a randomized controlled clinical trial showed that the Mediterranean diet could significantly improve HDL and effectively mitigate both low-density lipoprotein (LDL) and TG.^27^ Properzi *et al*.^28^ found that the Mediterranean diet group ameliorated liver steatosis and improved total cholesterol and serum TG levels but reported no significant changes in LDL and HDL. MedDietScore is a score to evaluate adherence to the Mediterranean diet. In a case-control study, Kontogianni *et al*.^29^ discovered that MedDietScore was negatively correlated with serum alanine aminotransferase (ALT) and insulin levels, insulin resistance index, and steatosis severity after adjusting for sex and abdominal fat level. A one-unit increase in MedDietScore was associated with a 36% reduction in the incidence of NASH.

**Fig. 2:**
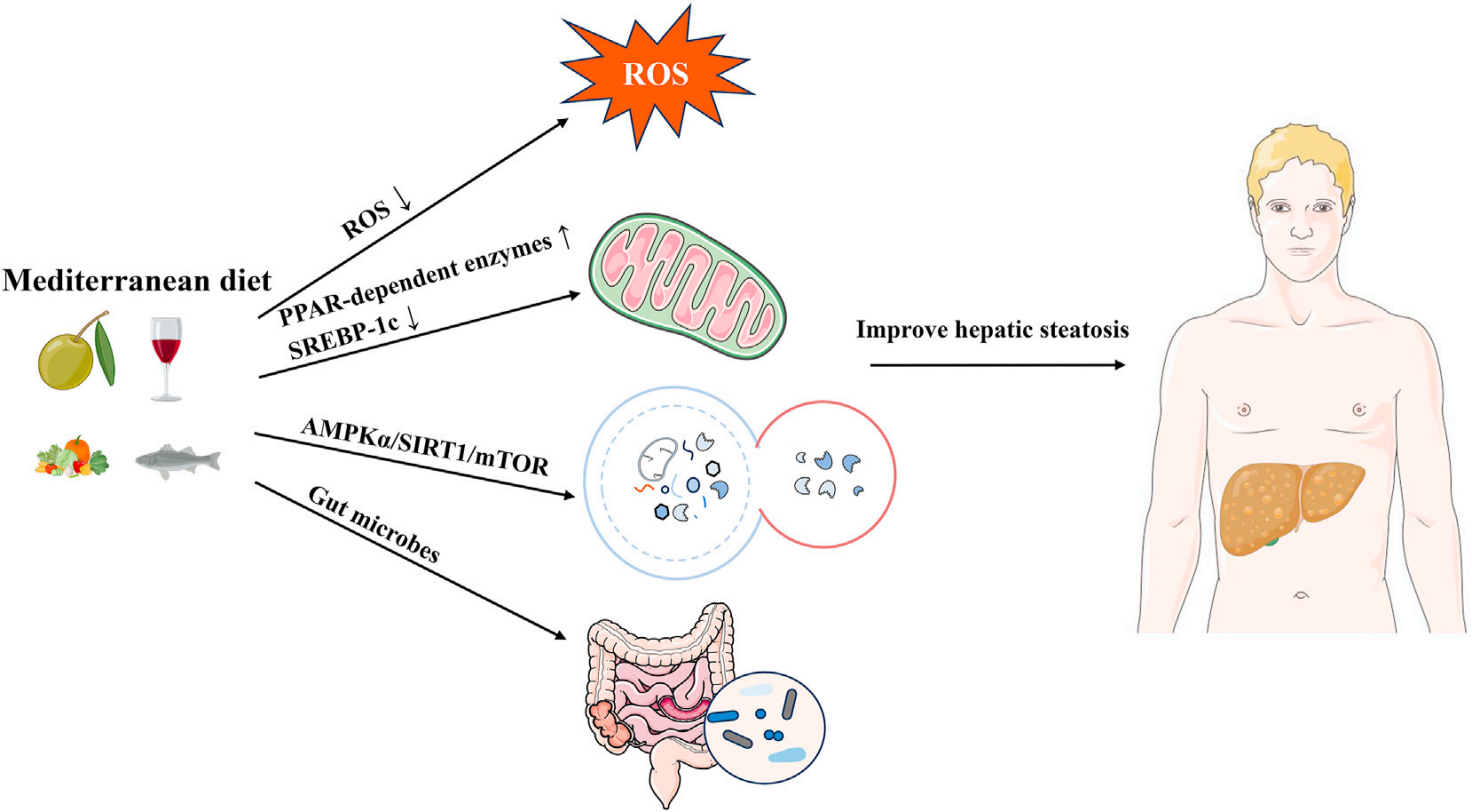
**Key molecular mechanisms of the Mediterranean diet in hepatic lipid metabolism.** The Mediterranean diet regulates the production of reactive oxygen species (ROS), peroxisome proliferator-activated receptor (PPAR)-dependent enzymes, sterol regulatory element-binding protein-1c (SREBP-1c), AMP-activated protein kinase alpha (AMPKα), Sirtuin1 (SIRT1), the mammalian target of rapamycin (mTOR), and the gut microbiome. This diet improves hepatic steatosis by ROS, fatty acid oxidation, autophagy, and gut microbiome.

Although discussing the role of a single component in the dietary pattern differs from the aim of this review, olive oil, a very characteristic food of the Mediterranean diet, is a significant study topic. In the Mediterranean region, it is often emphasized that the high intake of vegetables is closely linked to the use of olive oil because it can improve the texture and taste of food.^30^ It is crucial to note that the olive oil mentioned in the Mediterranean diet is “virgin olive oil,” not ordinary “olive oil”; this distinction may not be widely known outside the Mediterranean region. Virgin olive oil is obtained entirely via mechanical means from the fruit of the olive tree without any additional procedure, including washing, decanting, centrifugation, and filtration.^31^ Virgin olive oil contains many healthy compounds and is therefore considered a functional food by the European Food Safety Authority.^32^ The main difference between virgin olive oil and olive oil lies in its high content of antioxidants, including carotene and bioactive phenols.^33^ Both are rich in monounsaturated fatty acids (MUFAs), which possess antioxidant and anti-inflammatory activities.^34^ The occurrence and development of NAFLD are due to various factors, and oxidative stress is considered a key factor.^35^^,^^36^ Elevated levels of reactive oxygen species (ROS) can regulate the activity of the insulin signaling pathway, affect the expression and activity of lipid metabolism-related enzymes, and ultimately lead to a redox-dependent imbalance of hepatic lipid metabolism.^37^ The antioxidants in the Mediterranean diet can potentially impact the progression of NAFLD by modulating ROS levels (Fig. 2).^38^ In addition, some studies investigating the changes of fat in liver transcriptome have shown increased expression of many peroxisome proliferator-activated receptor (PPAR)-independent enzymes mediating fatty acid oxidation and decreased expression of genes related to sterol regulatory element-binding protein-1c (SREBP-1c) and lipid synthesis after olive oil administration (Fig. 2).^39^^,^^40^ These findings prove that liver TGs and fat synthesis are reduced in the Mediterranean diet.

Autophagy is a highly conserved cellular process that is crucial in degrading damaged cellular components under stress conditions, providing energy to stressed cells.^41^^,^^42^ Both the cellular level and animal models have shown that impaired autophagy in the liver is associated with NAFLD, as has been validated in those with NAFLD.^43^, ^44^, ^45^ Diet can regulate autophagy, which is related to autophagy activation.^46^ However, more comprehensive research is needed on the direct impact of the entire Mediterranean diet on autophagy. Moreover, researchers are still studying the effects of certain substances in the Mediterranean diet on autophagy. Resveratrol, a phenol in grapes, wine, and nuts, is thought to increase the activity of deacetylase Sirtuin1 (SIRT1), which in turn induces autophagy.^47^^,^^48^ Additionally, oleuropein (OLP), a polyphenol found in virgin olive oil, is an autophagy inducer that regulates the AMP-activated protein kinase alpha (AMPKα)/SIRT1/mammalian target of rapamycin (mTOR) pathway.^49^, ^50^, ^51^ OLP positively regulates autophagy by activating AMPK and inhibiting mTOR.^52^^,^^53^ These are the effects of the Mediterranean diet on liver fat metabolism through autophagy (Fig. 2). The above findings suggest the effects of the Mediterranean diet on hepatic lipid metabolism through autophagy. Nonetheless, further research is warranted to explore the comprehensive impact of autophagy on fat metabolism concerning the complete Mediterranean diet.

Various dietary patterns have been shown to impact the variety and function of gut microbes, which in turn affects organ metabolism.^54^, ^55^, ^56^ From the single component of the Mediterranean diet, MUFA is thought to increase the proliferation of the *Clostridiales vadin* BB60 group, while polyunsaturated fatty acid (PUFA) is believed to increase *Bifidobacterium*, *Lactobacillus*, and *Roseburia*.^57^, ^58^, ^59^ The complete Mediterranean diet has also been shown to alter the variety and abundance of gut microbes.^60^^,^^61^ The Mediterranean diet can promote beneficial alterations in the gut microbiota by reducing Firmicutes and increasing Bacteroides, which can improve obesity, inflammation, and related metabolic alterations.^62^ One randomized controlled trial (RCT) showed that the Mediterranean diet lowers serum cholesterol and increases insulin sensitivity by increasing levels of *Faecalibacterium prausnitzii* and microbial carbohydrate-degrading genes associated with butyric acid metabolism.^63^ Godny *et al*.^64^ demonstrated that a four-week Mediterranean diet intervention could increase the absolute abundance of multiple beneficial intestinal commensal bacteria, such as *Faecalibacterium* and *Lachnospira*. Despite numerous studies exploring the impact of the Mediterranean diet on gut microbes and the association between gut microbes and NAFLD, direct research that comprehensively combines all three aspects is currently lacking. Further investigation regarding this area would provide valuable insights into the intricate interplay between the Mediterranean diet, gut microbes, and NAFLD.

## 3 The Dietary Approaches to Stop Hypertension (DASH) diet and hepatic lipid metabolism

The DASH diet was initially proposed to prevent and treat high blood pressure in 1997.^65^ This diet is characterized by lower sodium intake, lower saturated fat, and higher levels of fiber and potassium compared to other diets.^66^ It emphasizes the intake of fruits, vegetables, and low-fat dairy products while limiting the consumption of saturated and total fat.^65^^,^^67^ A systematic review and Meta-analysis of 30 RCTs found that the DASH diet reduces blood pressure, particularly in people with daily sodium intake >2400 mg,^68^ in agreement with other studies.^66^^,^^69^^,^^70^ Recent studies have also shown that the DASH diet is associated with reduced all-cause mortality, cardiovascular morbidity or mortality, cancer morbidity or mortality, type 2 diabetes, and neurodegenerative disease incidence.^71^^,^^72^

The DASH diet has a vital impact on hepatic lipid metabolism. Notably, it has demonstrated considerable efficacy in promoting weight loss compared to regular diets and even other low-calorie diets, with additional benefits observed in reducing abdominal fat.^66^^,^^73^ A Meta-analysis of 17 studies revealed that the DASH diet substantially reduced serum TG and LDL levels but did not change HDL and total cholesterol levels.^74^ This finding aligns with another Meta-analysis that supported the positive impact on TGs and LDL levels.^75^ However, some studies reported different results. Ge *et al*.^66^ found no significant changes in LDL and HDL levels in the population. For a more comprehensive understanding, future larger-scale prospective studies with fewer confounding factors could further elucidate the effect of the DASH diet on lipid profiles. For patients with NAFLD, the DASH diet improves weight, body mass index (BMI), ALT, alkaline phosphatase (ALP), TGs, markers of insulin metabolism, inflammatory markers, glutathione (GSH), and malondialdehyde (MDA).^76^ Additionally, a case-control study identified an inverse association between the DASH diet and NAFLD risk.^77^

Cereal fiber in the DASH diet is believed to stimulate satiety, leading to weight reduction. Additionally, the fiber that enters the stomach can slow down gastric emptying and glucose absorption, thereby reducing the insulin response and feelings of hunger.^78^^,^^79^ At the same time, DASH emphasizes a reduction in fat intake, including saturated fatty acid and total fat.^65^ These are in control of total energy intake and subsequently decrease fat accumulation in the liver. Hepatic steatosis is closely related to systemic insulin resistance, which can stimulate lipogenic enzymes through SREBP-1c, promoting *de novo* synthesis of fatty acids.^80^ Moreover, insulin resistance can cause increased free fatty acids from adipose tissue lipolysis, ultimately leading to NAFLD.^81^^,^^82^ Epidemiological studies have shown an inverse association between adherence to the DASH diet and insulin resistance.^83^ However, molecular-level research to explain the mechanisms behind this correlation is lacking. Similar to the Mediterranean diet, the DASH diet includes a significant amount of vegetables and fruits, which are rich sources of antioxidant and anti-inflammatory molecules. These components could also contribute to the diet's potential benefits on hepatic metabolism.

In summary, research on the effects of the DASH diet on hepatic metabolism has primarily focused on epidemiological studies, with fewer mechanistic studies than the Mediterranean diet, leaving room for further exploration and understanding of the molecular pathways involved.

## 4 Ketogenic diet and hepatic lipid metabolism

Low carbohydrates, low protein, and high fat characterize the ketogenic diet.^84^ The history of this diet dates back to 1921 when fasting was initially discovered to be effective in treating epilepsy.^85^ Acetone and beta (β)-hydroxybutyric acid were found to accumulate in fasting populations.^86^ Scientists also found that this accumulation can also occur with diets that have high fat and low carbohydrates. Wilder began recommending the ketogenic diet for patients with epilepsy.^87^ Many studies have demonstrated the effectiveness of this diet for epilepsy treatment.^85^ However, new antiepileptic drugs with fewer side effects and improved adherence have led to the reduced use of the ketogenic diet for epilepsy treatment. In recent years, the ketogenic diet has regained the attention of researchers due to its potential efficacy in other diseases, such as cancer treatment. Some recent studies have indicated that the diet may inhibit tumor growth and enhance the effectiveness of radiotherapy.^88^^,^^89^

Similar to the previously mentioned diet patterns, the ketogenic diet is considered a practical approach to manage obesity and weight loss.^90^^,^^91^ The ketogenic diet differs from other diets in that it is high in fat, which means that people who adopt this dietary pattern have a different lipid metabolism. The ketogenic diet is associated with increased LDL,^92^ which is linked to increased CVD risk. Therefore, further research is needed to fully understand the long-term effects of the ketogenic diet on cardiovascular health. On the positive side, the ketogenic diet has demonstrated beneficial effects on the liver. A low-carb, high-fat ketogenic diet can reduce serum insulin levels and increase systemic fatty acid oxidation and ketogenesis.^93^^,^^94^ Thus, the ketogenic diet has been shown to reduce hepatic fat content.^95^ Additionally, the ketogenic diet also affects cholesterol synthesis. Decreased serum insulin level affects β-hydroxy β-methylglutaryl-CoA reductase activation, which may inhibit cholesterol synthesis.^96^

**Fig. 3:**
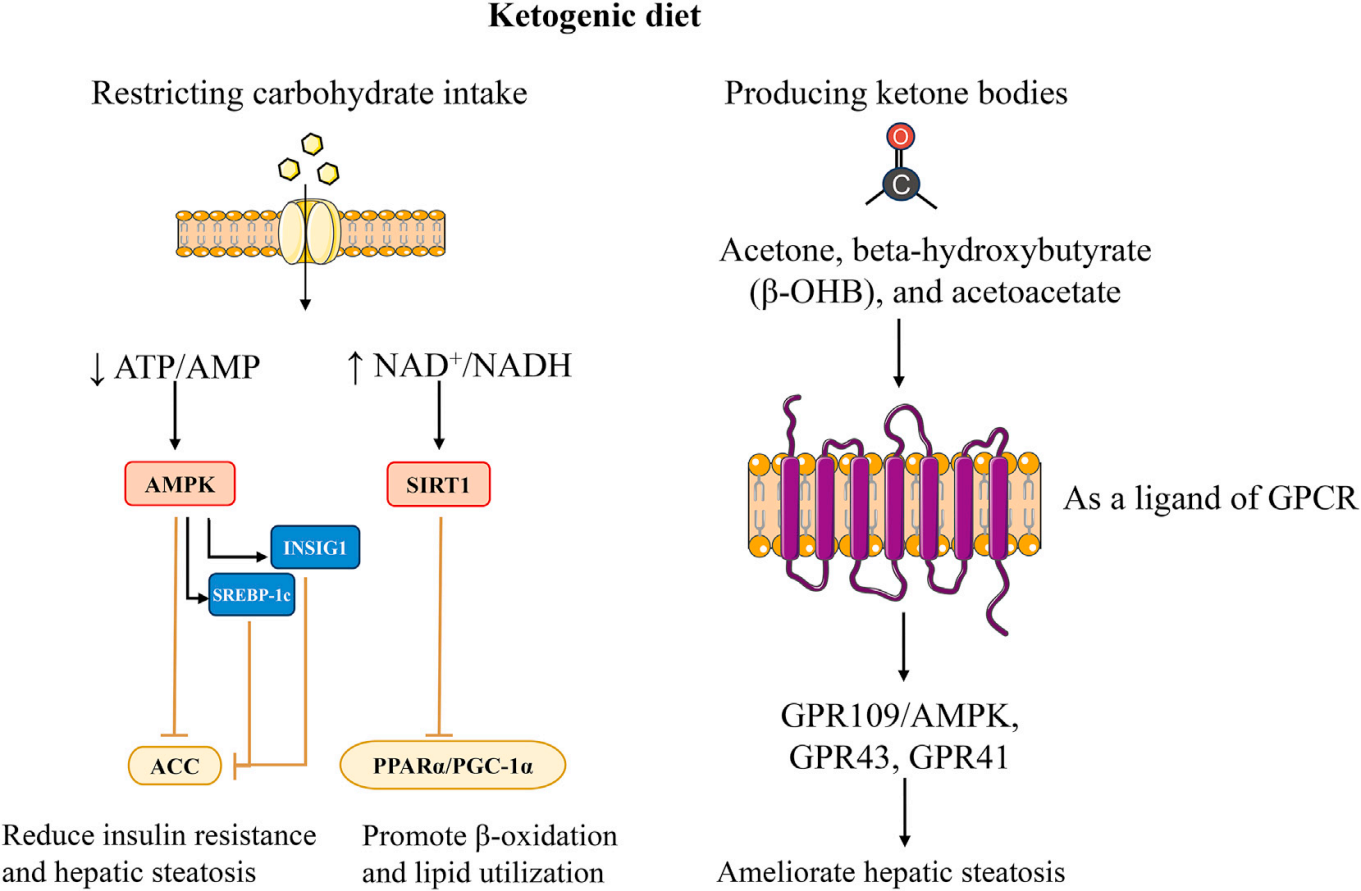
**Mechanisms of the ketogenic diet in the modulation of hepatic fat metabolism.** This figure depicts the ketogenic diet regulating fat metabolism by restricting carbohydrate intake and ketone body production. This includes the activation of AMP-activated protein kinase alpha (AMPKα), Sirtuin1 (SIRT1), and G-protein-coupled receptors (GPCRs). Abbreviations: ACC, acetyl-CoA carboxylase; AMP, adenosine monophosphate; ATP, adenosine triphosphate; INSIG1, insulin-induced gene 1; NAD^+^, nicotinamide adenine dinucleotide; NADH, nicotinamide adenine dinucleotide hydrogen; PGC-1α, peroxisome proliferator-activated receptor gamma coactivator-lalpha; PPARα, peroxisome proliferator-activated receptor alpha; SREBP-1c, sterol regulatory element-binding protein-1c.

The effect of the ketogenic diet on hepatic lipid metabolism is mainly achieved by limiting carbohydrate intake and ketone bodies (Fig. 3). On the one hand, restricting carbohydrate intake can reduce insulin levels, increase fatty acid oxidation rates, and decrease fatty acid synthesis.^97^ On the other hand, it can activate SIRT1 and AMPK pathways.^98^^,^^99^ The AMPK signaling pathway is considered a sensor of the cellular energy state, which is activated by energy stress through rising AMP and/or ADP coupled with falling ATP, thereby regulating the metabolic state to maintain energy homeostasis.^100^, ^101^, ^102^, ^103^ AMPK activation inhibits acetyl-CoA carboxylase (ACC), reducing insulin resistance and hepatic steatosis.^104^, ^105^, ^106^ AMPK can also transcriptionally inhibit ACC by phosphorylating SREBP-1c and insulin-induced gene 1 (INSIG1).^107^^,^^108^ SIRT1 is an essential metabolic/energy sensor that directly couples cellular metabolic/energy states (via intracellular NAD^+^/NADH ratios) to molecules involved in metabolic homeostasis, such as SREBP-1c and PPARα.^109^, ^110^, ^111^ SIRT1 deacetylates SREBP-1c, inhibiting lipogenesis and ameliorating hepatic steatosis.^112^^,^^113^ In addition, carbohydrate response element-binding protein (ChREBP) is another major transcription factor involved in lipogenesis.^114^ The histone upstream of the ChREBP promoter is deacetylated by SIRT1, controlling hepatic lipid homeostasis.^115^ SIRT1 can also promote β-fatty acid oxidation and improve lipid utilization through PPARα/PGC-1α.^111^

Ketone bodies include beta-hydroxybutyrate (β-OHB), acetoacetate, and acetone. The liver produces most ketone bodies as energy supplies under insufficient energy, especially to provide energy to the brain. In addition to energy supply, ketone bodies can serve as regulatory signals for cellular metabolic pathways.^116^ G-protein-coupled receptors (GPCRs) are signal-transduction proteins located on the plasma membrane of eukaryotic cells.^117^ GPCR interacts with G proteins in the plasma membrane after binding to the ligand to produce cAMP and other second messengers, which transduce cellular responses by regulating cell metabolism.^118^ Recent studies have shown that ketone bodies can act as ligands for GPCRs, regulating cell metabolism.^119^ GPR109A is a GPCR that is abundant in fat cells, macrophages, and neutrophils.^120^ In fat cells, GPR109A is activated by β-OHB, which reduces cAMP, decreasing the activity of hormone-sensitive lipase and inhibiting lipolysis.^121^ This mechanism is thought to be the negative feedback mechanism for ketone body synthesis.^122^ A recent study showed that β-OHB can inhibit hepatic lipid accumulation via the GPR109A/AMPK pathway in aged rats.^123^ Additionally, β-OHB can directly regulate sympathetic nerves to control energy balance through GPR41,^124^ but the specific mechanism is unknown. Another ketone body, acetoacetate, can activate lipoprotein lipase via GPR43 and improve plasma lipid utilization.^125^

In addition to the above mechanisms, the ketogenic diet can activate PPARα through fibroblast growth factor 21 (FGF21), promoting β oxidation of fatty acids.^126^ The ketogenic diet can also reduce fatty acid synthesis by inhibiting stearoyl-CoA desaturase activity.^127^ Indeed, there is a robust theoretical basis supporting the use of ketogenic diets in the management of patients with NAFLD.

## 5 Low-fat diet and hepatic lipid metabolism

The fat content of food affects the level of serum fatty acids, and high serum fatty acid levels affect the lipid metabolism of the liver, which leads to NAFLD. A low-fat diet aims to maintain the fat content at 25%–35%, while the rest of the energy is mainly provided by carbohydrates.^128^ Unlike the previously mentioned dietary patterns, low-fat diets are not always good. Shan *et al*.^129^ show that a healthy low-fat diet negatively correlates with overall mortality. In contrast, an unhealthy low-fat diet positively correlates with overall mortality, suggesting that the association between a low-fat diet and mortality may depend on the quality of macronutrients and food sources.^129^ Saturated or unsaturated fats may be key in differentiating healthy and unhealthy low-fat diets.

Recent studies have also demonstrated the positive effects of low-fat diets on lipid metabolism. Hansen *et al*.^130^ confirmed that a low-fat diet can reduce weight, lower LDL, and increase HDL. Other studies have reached similar conclusions.^131^^,^^132^ Animal studies have also confirmed that a low-fat diet can ameliorate histological liver lesions in NAFLD.^133^ These positive studies may have used a healthy, low-fat diet. There are also unhealthy low-fat diets, which are worth studying. Developing guidelines to distinguish between healthy and unhealthy low-fat diets is important.

Unfortunately, research on the impact of a low-fat diet on liver fat metabolism is scarce. However, it is known that in a low-fat diet, the intake of total fat and saturated fatty acids (SFAs) is reduced, and SFAs are harmful to the human body. Studies have shown that SFAs can induce sterile inflammation, one of the main causes of insulin resistance in organs.^134^ At the cellular level, the researchers demonstrated that SFAs weaken the insulin signaling pathway through Toll-like receptor 4 (TLR4) and that this inhibition can be eliminated by knocking out or inhibiting TLR4.^135^^,^^136^ Moreover, SFAs have greater lipotoxicity than MOFA and can promote NAFLD development through endoplasmic reticulum stress, mitochondrial metabolic imbalance, and the activation of Janus kinase (JAK) stress pathways.^137^ The low-fat diet reduces the intake of the overall fat intake, as well as SFAs, avoiding the risks caused by SFAs. However, it is still crucial to explore the lipid metabolism of the liver in the low-fat state and provide suggestions for the diets of those with NAFLD.

## 6 High-protein diet and hepatic lipid metabolism

People usually treat obesity by adjusting their diet patterns, but there is no standard answer. A high-protein diet differs from a low-fat, low-carb diet because 25%–35% of the daily energy comes from protein, and the proportion of energy from carbohydrates is less than 45%.^138^ According to recent studies, a high-protein diet has better effects on obesity and diabetes than a traditional low-fat diet.^139^, ^140^, ^141^ A randomized trial treating obesity showed that the high-protein diet group with 25% protein lost more weight and fat than the 12% protein diet.^140^ In addition, Larsen *et al*.^142^ demonstrated that a high-protein diet helps maintain weight loss. For the lipid profile, a high-protein diet did not significantly improve blood lipid levels and insulin sensitivity in participants with overweight or obesity and prehypertension or stage 1 hypertension without type 2 diabetes mellitus.^143^ For patients with severe obesity undergoing bariatric surgery, there was no significant difference in weight change between the high- and low-protein diet groups, but there was a more considerable decrease in intrahepatic fat in the high-protein diet group.^144^ This healthy mechanism of a high-protein diet may be related to the feeling of fullness after high protein, reducing food uptake.^145^^,^^146^ At the molecular level, it may be related to fat uptake and synthesis.^144^

Krüppel-like factors (KLFs) are a subclass of zinc DNA-binding transcription factors involved in various biological processes such as cell metabolism, cell proliferation, and inflammation.^147^^,^^148^ KLF15 is a KLF that is abundantly expressed in the liver and is involved in regulating glucose homeostasis, lipid metabolism, and amino acid synthesis.^149^, ^150^, ^151^ Studies have shown that high-protein diets adjust changes in liver metabolism through KLF15.^152^ Moreover, several studies have shown that KLF15 is associated with lipid metabolism.^153^^,^^154^ However, the effect of KLF15 on fat metabolism remains controversial.^151^ Studies indicate that leucine concentrations are significantly higher in animals on a high-protein versus low-protein diet, and leucine can stimulate mTOR pathway activation.^152^ As mentioned earlier, mTOR inhibition reduces hepatic steatosis, indicating that high-protein diets may not reduce intrahepatic fat content through mTOR. Specific mechanisms need to be explored. However, mTOR is activated in high-protein diets and is thought to be one reason that high-protein diets increase CVD risks.^155^

## 7 Other dietary patterns

Other dietary patterns, such as plant-based diets, have also been shown to be associated with fat metabolism. Plant-based diets involve reducing or restricting food of animal origin and increasing the intake of plant-based foods. A Meta-analysis of 11 RCTs demonstrated that total cholesterol, LDL, and HDL were significantly reduced after vegetarian diets, but TG level was not significantly changed compared to an omnivorous diet.^156^ Other studies have demonstrated the positive effects of plant-based diets on weight and obesity.^157^^,^^158^

Not all dietary patterns are beneficial for health. The Western diet, which is prevalent in modern Western society, is characterized by refined foods high in simple sugars, salt, SFAs, and cholesterol while lacking fiber and unsaturated fatty acids.^159^ A prospective cohort study in adolescents revealed a positive association between Western diets and NAFLD development.^160^

## 8 Conclusions and future perspectives

From an epidemiological perspective, there is evidence that dietary patterns influence hepatic lipid metabolism, leading to improvements in serum lipid profiles, weight control, and hepatic steatosis. The effects of each dietary pattern on hepatic lipid metabolism are summarized in Table 2. Various dietary patterns exist, allowing individuals worldwide to choose suitable options based on local food resources. The Mediterranean diet is currently considered the most appropriate dietary pattern for patients with NAFLD. This diet exerts its effects through oxidative stress, autophagy, and gut microbiota modulation. However, a noteworthy challenge in promoting the Mediterranean diet is the limited availability of key components like extra virgin olive oil outside the Mediterranean region. Additionally, while many diets can improve hepatic steatosis, different diets may carry different risks. For example, high-protein diets may predispose to CVD. These factors highlight the need for individualized dietary strategies based on available food resources, economic conditions, and underlying patient conditions.

**Table 2:** The effects of each dietary pattern on hepatic lipid metabolism.

Dietary patterns	Effects on hepatic lipid metabolism	References
Mediterranean diet	Reduce hepatic fat content, liver stiffness, TG level, and the incidence of NASH Increase HDL level	^26^^,^^27^^,^^29^
DASH diet	Reduce abdominal fat, TG level, LDL level, and the risk of NAFLD No change in HDL	^66^^,^^73^^,^^77^
Ketogenic diet	Reduce hepatic fat content and inhibit cholesterol synthesis Increase LDL level, fatty acid oxidation, and ketogenesis	^92^^,^^95^^,^^96^
Low-fat diet	Lower LDL level Increase HDL level Ameliorate NAFLD lesions in animals	^130^^,^^133^
High-protein diet	Reduce serum lipid level and hepatic fat content	^143^^,^^144^

Abbreviations: DASH, Dietary Approaches to Stop Hypertension; HDL, high-density lipoprotein; LDL, low-density lipoprotein; NAFLD, non-alcoholic fatty liver disease; NASH, non-alcoholic steatohepatitis; TG, triglyceride.

Furthermore, most clinical trials on dietary patterns in the past have been retrospective studies, which are prone to confounding factors. Future research should focus on larger prospective clinical trials to investigate the impact of dietary patterns on lipid metabolism. Additionally, there is a lack of research on the mechanisms by which different dietary patterns affect hepatic fat metabolism, with most studies concentrating on the Mediterranean and ketogenic diets. Future researchers should expand their investigations to include other dietary patterns such as the DASH, low-fat, and high-protein diets.

## Authors’ contributions

Lin Wang conceived the idea. Peng Zou wrote the manuscript and drew the figures. Lin Wang reviewed and revised the manuscript critically. Both authors read and approved the final manuscript.

## Declaration of competing interest

The authors declare that they have no conflict of interest.
